# Whole-Exome Sequencing for Molecular Diagnosis of Paediatric Nephrotic Syndrome in Africa: A Call for Implementation

**DOI:** 10.3390/genes16111295

**Published:** 2025-10-31

**Authors:** Thina Gcobo, Jonathan N. Katsukunya, Lindie Lamola, Denis Awany, Arinao Ndadza, Collet Dandara, Khuthala Mnika

**Affiliations:** 1 Division of Human Genetics, Department of Pathology, Institute of Infectious Disease and Molecular Medicine, Faculty of Health Sciences, University of Cape Town, Cape Town 7935, South Africa; thnthi002@myuct.ac.za (T.G.); ktsjon001@myuct.ac.za (J.N.K.); collet.dandara@uct.ac.za (C.D.); 2 Platform for Pharmacogenomics Research and Translation, South African Medical Research Council, Cape Town 7935, South Africa; 3 Division of Human Genetics, National Health Laboratory Service, School of Pathology, Faculty of Health Sciences, University of the Witwatersrand, Johannesburg 2193, South Africa; lindie.lamola@wits.ac.za; 4 South African Tuberculosis Vaccine Initiative, Institute of Infectious Disease and Molecular Medicine, Division of Immunology, University of Cape Town, Cape Town 7935, South Africa; denis.awany@uct.ac.za; 5 Department of Molecular Medicine and Haematology, School of Pathology, Faculty of Health Sciences, University of the Witwatersrand, Johannesburg 2193, South Africa; arinao.ndadza@wits.ac.za

**Keywords:** Africa, genetic testing, nephrotic syndrome, pathogenic variants, WES

## Abstract

Nephrotic syndrome (NS) is a common type of kidney disease in children, marked by protein loss in urine, swelling, and low blood protein levels. It is more severe and prevalent in children of African descent, particularly in steroid-resistant forms. Many cases are primary and linked to mutations in genes such as *NPHS1*, *NPHS2*, and *WT1*. While whole-exome sequencing (WES) has advanced the identification of genetic causes globally, its application in African settings remains limited, leaving many cases undiagnosed. This review explores the potential of WES in improving NS diagnosis among African paediatric populations. A literature search was conducted using PubMed, Scopus, and Medline for studies published between 2015 and 2025 focusing on the application of WES in paediatric NS among individuals of African descent. From the 12 articles retrieved, three met the inclusion criteria. These publications reported variants in *NPHS1*, *NPHS2*, *WT1*, *PLCE1*, *COL4A3*, *COL4A5*, *TRPC6*, and *LAMB2* among South African and Egyptian cohorts. WES remains underutilised in African NS research, hindered by limited resources, cost, and underrepresentation in genomic databases. Nonetheless, preliminary evidence suggests WES may contribute to improving diagnosis and guiding treatment through the identification of population-specific pathogenic variants. Increased investment in genomic infrastructure is important for maximising potential benefits and improving diagnostic capabilities.

## 1. Introduction

Nephrotic syndrome (NS), a type of glomerular chronic kidney disease (CKD), is characterised by a group of symptoms which include proteinuria (≥1000 mg/m^2^ per day), lipiduria, oedema and/or hypoalbuminemia to (<3g/dL) [1,2,3]. These symptoms arise because of impaired kidney function which limit the kidney’s filtering capacity. NS is one of the most common CKD and a major cause of kidney failure in paediatric patients [4]. NS has an estimated global incidence rate of 2–7 cases per 100,000 paediatrics [2], with a prevalence that is three times higher in African populations than non-African populations [5,6]. In Africa, the burden of NS differs by region. Tropical Africa has the highest incidence, accounting for up to 1.35% of hospital admissions [7,8]. Despite the limited studies in Southern Africa, it still appears to have one of the highest proportions of NS, with a prevalence rate of ~33% [7,8].

NS can be classified into two broad categories, primary and secondary, depending on the aetiology of the disease. Primary NS is the most common glomerular disease whose underlying aetiology is largely unknown, mostly diagnosed in children, and accounts for more than 90% of all cases [9]. Primary NS emerges independently of associated extrarenal diseases such as systemic lupus erythematosus but may result from multiple histopathological features such as minimal change disease (MCD), focal segmented glomerulosclerosis (FSGS), and membranous nephropathy (MN) (Table 1) [10].

Variations in genes coding for components of the glomerular filtration barrier have been reported to cause the histopathological features observed in primary NS. These genes include, but are not limited to, nephrin (*NPHS1*), phospholipase C epsilon 1 (*PLCE1*), laminin subunit beta 2 (*LAMB2*), podocin (*NPHS2*), and Wilms Tumour 1 (*WT1*) [11]. Approximately two-thirds of NS patients possess mutations in these genes suggesting that genetic factors play a significant role in hereditary forms of primary NS [11,12]. Furthermore, research conducted on NS across various populations and countries has strongly indicated a potential genetic predisposition to NS among certain ethnic groups [13]. According to the literature, Americans and Hispanics continue to present the most severe cases of NS [6], while in Africa, MCD and FSGS are the most prevalent forms of NS, with FSGS having the worst progressive outcomes [2].

The proportion of patients with steroid resistant NS (SRNS) or adverse forms of NS seems to be higher in Asians (27 to 54%) and Africans (16 to 73.5%) compared to individuals of European ancestry (20%) [5,7,14,15,16]. This means that more African children experience a significant decline in kidney function, and some even develop end-stage kidney disease (ESKD) before reaching the age of 10 years. Among Africans, there have been studies, although still limited, on the role of genetic susceptibility to NS, including SRNS. For example, two studies conducted in South Africa by Asharam et al. [13] and Nandlal et al. [17] report on higher proportions (95%) of SRNS in African children, with 33% and 21%, respectively, carrying the *NPHS2* NM_014625:c.779T>A (V260E) variant (which is strongly associated with increased disease severity) compared to non-African children (i.e., of Indian ancestry) who were burdened with a less severe form of NS, steroid sensitive NS (SSNS) [17]. Thus, the African genome may carry unique genomic elements, which predispose their carriers to increased susceptibility. However, given the genetic diversity among Africans, it appears there is more to be discovered in African genomes, particularly in the paediatric cases of NS (Table 1).

Post completion of the first draft of the Human Genome Project, next generation sequencing (NGS) technologies have revolutionised genomic research and diagnostics, including in paediatric NS. Technologies such as whole-genome sequencing (WGS), whole-exome sequencing (WES), and targeted gene panel testing have advanced identification of pathogenic variants across multiple genes associated with NS. Gene panel testing is cost-effective and well-suited for patients with typical phenotypes linked to known gene mutations. However, it may miss causal variants in genes not included in the panel. WES, although more expensive, provides broader coverage of the coding genome and can enhance diagnostic yield by detecting variants beyond those included in targeted panels, including novel gene-disease associations. WGS offers the most comprehensive genomic coverage and holds the potential to resolve cases undiagnosed by WES. Nevertheless, due to its high cost, WGS is typically reserved as a final option, particularly in resource-limited settings such as many regions in Africa.

**Figure 1:**
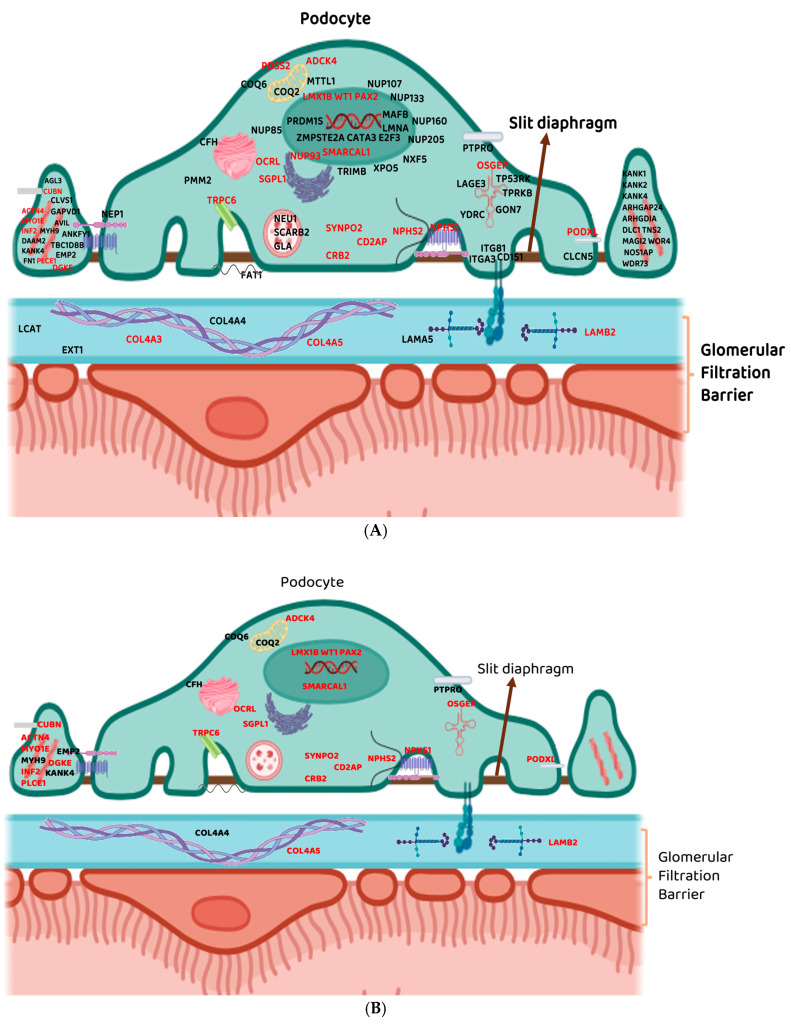
Glomerulus structure (podocytes and part of the glomerular filtration barrier) and genes involved in NS pathology. (**A**): Genes identified in Western countries and populations that are not of African descent. (**B**): Genes identified in studies using African-specific populations. Genes with variants identified through WES in African populations and were reported in Europe are highlighted in red. Genes not highlighted were screened in African populations using other sequencing methods. The evidence level of the identified gene–disease association was obtained from the Clinical Genome Resource (ClinGen) (http://www.clinicalgenome.org/) curated content; *COL4A5*, *DGKE*, *INF2*, *PLCE1*, *COQ8B/ADCK4*, *LMX1B*, *PAX2*, *OCRL*, *SGPL1*, *CRB2*, *NPHS1, CD2AP*, NUP93 and *COL4A3* have definitive associations. *TRPC6*, *MYO1E*: under review for curation. *NPHS2*, *WT1*, *SYNPO2* and *LAMB2:* in the pre-curation stage. *ACTN4*, *OSGEP*, *SMARCAL1*, *CUBN*, *PDSS2* and *PODXL*: in scope for pre-curation (Supplementary Table S1).

To date, WES has facilitated the discovery of over 50 genes and more than 80 variants linked to previously unresolved NS phenotypes (Figure 1). By applying WES and analysing 53 SRNS-associated genes, Bierzynska et al. [18] identified a monogenic cause in 26.6% of cases, confirming the presence of pathogenic variants within the SRNS cohort. Similarly, Trautmann et al. [19] achieved a 24% genetic diagnosis rate in SRNS cases by screening for known SRNS-related genes, while also identifying novel variants in genes not previously implicated in the disease. Additionally, a link was established between genetically confirmed SRNS and an increased risk of developing ESKD in childhood [19]. These findings could aid in pre-screening NS patients to investigate the presence of pathogenic variants within these genes before initiating treatment. Expanding the analysis to include recently discovered SRNS-associated genes might explain some of the remaining cases [18]. Nonetheless, these studies were able to present crucial steps in patient management and predicting disease progression [18,19].

NGS utility has been beneficial for non-African populations, such as American, Asian, and European populations, to such an extent that the incorporation of genetic testing through WES has become standard of care for NS in most Western countries [19]. This is, however, still not the case for Africa as the utility of NGS is still not widespread, particularly for paediatric NS. As such, its diagnostic potential and ability to resolve even complex NS phenotypes, as demonstrated in non-African populations, has not been realised for African populations largely due to the lack of studies in African populations [20]. This has been a limitation for Africa as this lack of understanding and evidence of WES utility within the continent might be restricting its incorporation in molecular diagnosis of African paediatric patients, leading to many unsolved NS cases. Therefore, this review aims to highlight the utility of NGS, particularly WES, in the diagnosis and management of NS within the African context, and to provide insights into its application to potentially improve clinical outcomes on the continent.

## 2. Methods

### 2.1. Literature Search Strategy

A comprehensive literature search was conducted using the online databases PubMed (National Library of Medicine), Scopus, and Web of Science. Published articles that either reported on the utilisation of NGS as a diagnostic tool for NS or were investigating the benefits of NGS in the field of paediatric nephrology were retrieved. The keywords used included the individual use or a combination of these Medical Subject Headings (MeSH) terms: “whole-exome sequencing” “whole genome sequencing”, “NGS gene panel sequencing”, “nephrotic syndrome”, “molecular diagnosis”, “Africa”, “paediatric patients”, together with their most relevant synonyms (i.e., complete exome sequencing, complete genome sequencing, high throughput nucleotide sequencing, paediatric idiopathic nephrotic syndrome, diagnostic molecular pathology and child/children). Boolean operators “OR” and “AND” were used to separate the synonyms and to string together the MeSH terms. Searches were restricted to research articles only, which could be accessed in full and written in English with a range from 2015 to March 2025.

### 2.2. Screening and Selection of Studies

The Preferred Reporting Items for Systematic Reviews and Meta-Analyses (PRISMA) 2020 [21] was adopted for this review. The initial screening of the retrieved studies was performed by T.G. to select studies with the following inclusion criteria: (1) studies utilising WES/ WGS/ NGS gene panel sequencing in paediatric (neonates to 19 years) NS (or related phenotypes) and (2) studies where the participants were of African descent (defined as having an ancestral trace to Africa) or conducted in Africa. The exclusion criteria were (1) studies with adult participants or non-paediatric patients (older than 18 years) only, (2) studies that did not include African participants even if they were conducted in Africa, and (3) studies using targeted genetic characterisation methods (e.g., targeted Sanger sequencing), review articles, letters, editorials, notes, incomplete, abstracts without full texts, and those irrelevant to the scope. Studies older than 10 years were also excluded from the review. All authors independently verified the screening of articles and checked the extracted data to avoid bias of selecting papers; this was performed between March and June 2025.

## 3. Results

**Figure 2:**
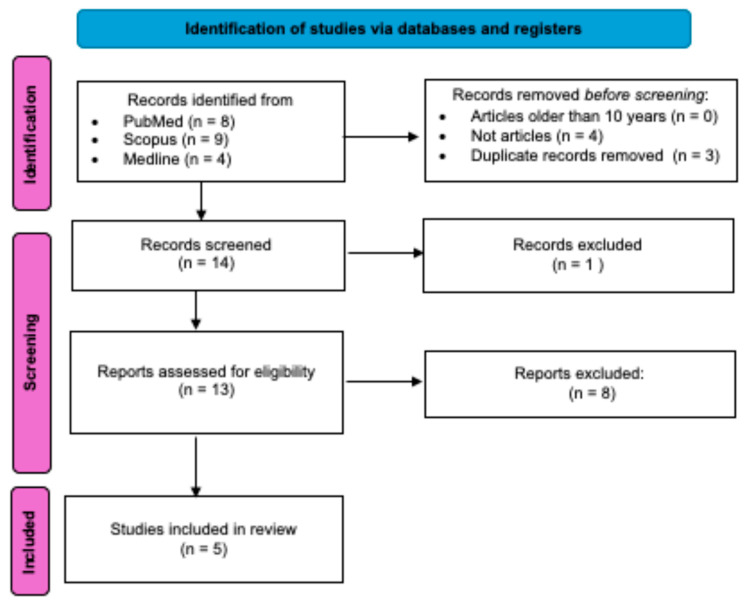
Selection process for studies identified across different databases and included in the review. The studies were excluded during screening because they either (1) included only adult participants (>18 years), (2) did not specify their study populations nor setting, and/or (3) used targeted genetic methods.

From the literature search, 21 citations were retrieved, eight from PubMed, nine from Scopus, and four from Medline. Prior to screening, seven citations were excluded because they were either duplicates (1) or were not research articles (2). This resulted in 14 research articles that were screened and assessed for eligibility. Nine research articles were excluded due to not meeting the inclusion criteria. Consequently, five studies were included in this review (Figure 2 and Supplementary Table S1), and the characteristics of these studies are shown in Table 2. The genes reported in these studies have been grouped according to (1) commonly reported genes across all the retrieved studies utilising NGS techniques and (3) genes specific to each retrieved study identified. These also detected novel variants in genes previously reported to associate with SRNS across the three studies, and these are in listed in Table 3. The novel variants detected were likely pathogenic, probably damaging or damaging, deleterious or disease causing, except inverted formin 2 (*INF2*) S409T and *PLCE1* A757E, which were benign or tolerated as classified by in silico prediction tools.

### 3.1. Commonly Reported Genes Across All the Retrieved Studies Utilising Next-Generation Sequencing Techniques

Though over 80 variants found in more than 50 genes that are associated with NS globally have been identified [18,27], only 13 genes have been identified in populations of African ancestry (Figure 1) [20]. However, in Africa, conventional genotyping techniques, primarily Sanger sequencing and polymerase chain reaction (PCR)-based techniques, have commonly been used to screen for pathogenic variants that have been previously reported to associate with NS, largely identified in non-African populations. These include variants in the commonly mutated ‘NS genes’: *NPHS1*, *NPHS2*, *WT1*, *LAMB2*, alpha-actinin-4 (*ACTN4*), transient receptor protein cation channel subfamily C member 6 (*TRPC6*), *INF2*, CD2 associated protein (*CD2AP*), *PLCE1*, and myosin E (*MYO1E*). However, the findings of these studies may not be fully generalisable and translated to the broader African context, given the continent’s high genetic diversity.

#### 3.1.1. *NPHS1* and *NPHS2*

Mutations occurring in the *NPHS1* gene, responsible for encoding the protein nephrin, result in steroid-resistant CNS, mostly in the Finnish population and less common in the non-Finnish populations [28,29,30]. On the other hand, mutations in the *NPHS2* gene, which encodes podocin, are linked to autosomal recessive SRNS in older children, across different ethnicities [31]. These genes interact to help maintain the integrity of podocytes and the glomerular filtration barrier [32,33]. Across the retrieved studies, the majority of the study participants had a high frequency of likely pathogenic or pathogenic variants in these genes (ranging between 6.7 and 60%) [17,18,22,23,24].

Evidently, in a South African study by [17], the most frequently mutated gene was *NPHS2*, with the *NPHS2 V260E* (reported as NM_014625.4:c.3032-21A>T) mutation found in a homozygous state in 21% of Black South African children with SRNS (n = 56), while it was absent in Indian patients. This variant (*V260E*) was also reported in a study conducted in France (1/10 patients with AR mutations), although they did not mention the ethnicity of the family in which the variant was identified [23]. Furthermore, no additional previously reported or novel pathogenic variants were detected in the *NPHS2* gene, and no mutations were identified in the *NPHS1* gene in this study [17]. Similarly, in Egyptian children, *NPHS2* was one of the most mutated genes, with mutations NM_014625.4:c.1A>T, NM_014625.4:c.467dupT, and NM_014625.4:c.502C>T detected [22,24]. Though not novel, an additional missense variant (NM_014625.4:c.890 C>T), classified as pathogenic, was detected in the study that performed WGS on Egyptian children [24]. In particular, *NPHS2* NM_014625.4;c.1A>T, previously reported as a founder mutation in the Egyptian population [34], was found in 57.1% patients with NS related to *NPHS2* and was implicated in two sibling cases with CNS, a phenotype of NS characterised by nephrotic-range proteinuria and oedema within the first three months after birth (Table 1) [22,24]. This variant (NM_014625.4;c.1A>T) was also identified in two families with SRNS in another Egyptian study [24] but was absent in none-Egyptian studies [17,18,23], emphasising its founder effect. In contrast to [17], seven *NPHS1* mutations were detected in the [22] study, and these variants were either frameshift, missense, or nonsense mutations. Six of these variants were likely pathogenic, except NM_004646.4:c.3478C>T, which was flagged as pathogenic according to the American College of Medical Genetics and Genomics (ACMG) guidelines [22].

NM_004646.4:c.3478C>T was also identified in the study conducted in France [23], which screened genes associated with SRNS to analyse their association with *APOL1* high-risk and low-risk genotypes. *NPHS1* was the most mutated gene in the low-risk genotype group with known familial cases (those without the combination of both a G1 and G2 risk allele), followed by *NPHS2* (3.29% participants and 1%, respectively, n = 152). Only one participant in the high-risk genotype group, who presented with CNS, had two compound heterozygous *NPHS1* mutations. Of the reported participants with the *NPHS1* variant (NM_004646.4:c.106 delG), one had extra renal manifestations, which include microcephaly and pulmonary valve stenosis, while the one with the *NPHS2* NM_004646.4:c.781G>T variant presented with pulmonary artery stenosis [23]. No other common variants within the *NPHS1* and *NPHS2* genes were identified.

In the study by [18], both the *NPHS1* and *NPHS2* genes were among the frequently mutated genes. The study included patients with diagnosed SRNS, with *NPHS2* mutations found in children older than two years, whilst *NPHS1* mutations were only identified in children with CNS and INS [18]. The *NPHS2* mutations present in 35% of the children older than two were either known to be pathogenic (NM_014625.4:c.413G>A, NM_014625.4:c.868G>A, NM_014625.4:c.686G>A, NM_014625.4:c.871C>T, and NM_014625.4:c.378+5G>A) or novel and predicted to be likely pathogenic (NM_014625.4:c.378+1_378+2delinsTG) [18] (Table 3). In the cohort with *NPHS1* variants, six novel disease-causing variants (NM_004646.4:c.158C>T, NM_004646.4:c.2387G>A, NM_004646.4:c.136G>T, NM_004646.4:c.925G>A, and NM_004646.4:c.1910_1912delTCT) were identified in 14 out of 15 patients with CNS and INS [18].

#### 3.1.2. *PLCE1* and *WT1*

*PLCE1* encodes the phospholipase C epsilon protein and is crucial for the development and function of podocytes. Mutations in this gene (exhibit autosomal recessive inheritance patterns) [35] have been associated with a disruption to the glomerular filtration barrier, resulting in FSGS CNS or early-onset NS. Three *PLCE1* variants NM_016341.4:c.689_690del, NM_016341.4:c.5363dup, and NM_016341.4:c.2779G>T were identified in Egyptian children and were classified as pathogenic and likely pathogenic, respectively [22,24] (Table 3). In some cases, these variants together with the mutations in the *NPHS1* and *NPHS2* were linked to manifestations beyond the kidneys [22]. Furthermore, the NM_016341.4:c.5363dup variant was found in one of the eight patients with FSGS and the only one who received complete remission for 2.3 years after being started on CsA treatment [22].

The *WT1* gene, responsible for encoding the transcription factor Wilms tumour suppressor 1, is involved in the formation and management of podocytes, and mutations in this gene have also been associated with early-onset NS, syndromic and non-syndromic NS, with a progression to ESKD [12]. Even though no pathogenic or likely pathogenic variants in *PLCE1* and *WT1* were identified in the [17] study, *WT1* was still one of the frequently mutated genes in the other four studies [18,22,23,24]. The *WT1* NM_024426.6:c.1432+5G>A variant was associated with CNS and was the second frequent mutation in the study group with an age onset below two years of age and was popular in patients with a familial SRNS [18] and in a participant who reached ESRD at age 9 [23]. Alternatively, the *WT1* NM_024426.6:c.1432+1G>A likely pathogenic splice (KST) variant was identified in a FSGS patient who also presented with Frasier syndrome, and in another patient with monogenic FSGS who achieved transient partial remission when started on cyclosporin A (CsA) therapy, a second-line calcineurin inhibitor [22]. The novel *WT1* variant NM_024426:c.700G>C (Table 3), although classified as a variant of uncertain significance (VUS) according to the ACMG guidelines, is considered by the authors, based on supporting evidence (AD inheritance patters, segregation analysis, and population frequencies), as a probable cause of the phenotype observed in the families studied. In this study, they reported one other *WT1* variant (NM_024426:c.1447+5G>A), which was not reported in the other studies and is classified as likely pathogenic [24].

### 3.2. Other Commonly Reported Gene Across All the Retrieved Studies Utilising Next-Generation Sequencing Techniques

#### 3.2.1. *COL4A3* and *COL4A5*

Variants in genes not usually associated with the development of podocytopathies like those in the *COL4A* genes have been of interest lately, as they have been linked to the development of secondary FSGS in adults [19,36]. *COL4A3*, *COL4A4*, and *COL4A5* genes encode the alpha chains of type IV collagen, a structural component of the glomerular basement membrane (GBM). In the study by [22], they identified homozygous *COL4A3* NM_000091.5:c.820_821delinsC and hemizygous pathogenic variants in *COL4A5* c.2732G>A (Table 3) within their infantile SRNS cases who presented with only mesangial proliferation as a histopathological feature. One patient who presented with SRNS has a mutation in the *COL4A3* gene, and this variant NM_000091.5:c.2621_2622delGAinsT has been associated with familial haematuria [18]. Furthermore, the study suggests that coinheritance of *COL4A5* and *MYO1E* variants predicts an increased severity of kidney diseases, and a novel *COL4A5* c.3097G>C variant was identified together with *MYO1E* variants in a patient with presumed nephropathy and no confirmed diagnosis [27]. Novel variants in the collagen genes have emerged as being specific to Egyptian and Tunisian populations [37,38], and more *COL4A* variants are emerging as main causes of SRNS in Asian children [36]. As such, a novel *COL4A3* (NM_000091.5: c.2126-1G>A) intronic variant classified as likely pathogenic was identified in an Egyptian family [24] (Table 3). These genes were not screened in South African children nor in the study conducted in France [17,23].

#### 3.2.2. *TRPC6*

*TRPC6* encodes a calcium-permeable ion channel protein which is expressed in the podocytes and is one of the components of the glomerular slit diaphragm. Mutations in this gene have been associated with autosomal dominant cases of familial FSGS. A single case of SR-FSGS (Indian-South African), with an age of onset of 18 years, was reported to have a novel probably damaging mutation (NM_004621:c.485G>T) in the *TRPC6* (Table 3) gene and achieved partial remission following second line of immunosuppressors [17]. Two sporadic infantile SRNS cases had mutations NM_004621:c.523C>T (novel) and NM_004621:c.2345A>T in the *TRPC6* gene (Table 3), which were both classified as potentially pathogenic [18]. Interestingly, none of the AD SRNS cases in had variants present in the *TRCP6* gene, which might mean *TRPC6* mutations might be a rare cause of SRNS in the Egyptian population [22,24]. The *TRPC6* variant NM_004621:c.2150 T>C was identified in one participant in this study, who presented as a sporadic case [23].

#### 3.2.3. *LAMB2*

Mutations in *LAMB2*, which codes for Laminin subunit beta-2 protein that is a component of the GBM, cause Pierson syndrome (a type of CNS). A novel likely pathogenic homozygous variant (NM_002292.4:c.5368C>T) and a previously reported VUS (NM_002292.4:c.1178_1180del) were identified in Egyptian children with Pierson syndrome, Galloway-Mowat syndrome, and insufficient adrenalin [22,24]. Likewise, a previously reported variant (NM_002292.4:c.736C>T) was identified in a patient with CNS Pierson syndrome [23,27].

### 3.3. Genes Specific to Each Retrieved Study Identified Using Next-Generation Sequencing Techniques

Of the screened genes through WES data analysis, [17] reported on identifying putative causal missense variants predicted to be probably damaging in the SRNS-FSGS cases, with the *INF2* ENST00000252527.8:c.1226G>C being more common than the *CD2AP* NM_012120.3:c.1898A>G. Reference [24] also reported a *CD2AP* VUS NM_012120.3:c.902A>T, though screened, none of the other studies report variants in these genes [18,22]. Elshafey et al. [22] screened for a panel of 27 SRNS-associated genes, and they found likely pathogenic and pathogenic novel variants in *OSGEP* (NM_002292.4:c.775A>T), *PAX2* (NM_003989.5:c.869del), and *SGPL1* (NM_003901.4:c.1013A>G) (Table 3), as well as previously reported likely pathogenic and pathogenic variants *CUBN* (NM_001081.4:c.8355delA), *PDSS2* (NM_020381.4:c.1145C>T), and *SYNPO2* (NM_133477.3:c.3370A>T). Nonetheless, these variants were associated with other NS-related syndromes as mentioned with the LAMB2 variants [22]. Additionally, novel or previously reported pathogenic or likely pathogenic variants in *ACTN4*, aarF domain-containing protein kinase 4 (*ADCK4*), crumbs cell polarity complex component 2 (*CRB2*), diacylglycerol kinase epsilon (*DGKE*), LIM homeobox transcription factor 1-beta (*LMX1B*), *MYO1E*, nucleoporin 107 (*NUP107*), nucleoporin 93 (*NUP93*), inositol polyphosphate-5-phosphatase (*OCRL*), and podocalyxin-like (*PODXL*) (Table 3) were also identified in cases with different NS phenotypes such as FSGS, nail patella-like kidney disease, and nephropathy [18]. Additionally, VUSs, likely pathogenic, or pathogenic variants in arachidonate 12-lipoxygenase, 12R type (*ALOX12B*), chromosome 12 open reading frame (*C12orf*), SNF2 related chromatin remodeling annealing helicase 1 (*SMARCAL1*), *PODXL*, *LMX1B*, *MYO1E*, and *NUP93* were also identified in Egyptian SRNS patients [24].

## 4. Discussion

The purpose of this review was to investigate the utilisation of WES to uncover and understand the genetic spectrum of NS in children of African descent. We found that not much has been performed in Africans in comparison to Asian and European populations [20], and that WES utility is still very limited. In a review conducted by [8], where they combined all NS studies conducted across Africa between 1963 and 2020 to highlight the epidemiology, frequencies, and histopathological features of the different types of NS, it was reported that the majority (69%) of African countries had no reported data on NS. Of the 81 paediatric NS studies conducted in 19 countries, South Africa contributed only 8.64% [7,8]. This shows that not only is there a limited understanding of NS genomics in Africa, but the problem is multifaceted, and the lack of rich epidemiological data across many African countries may be contributing to the overall lack of attention in this field.

Currently, the genomic characterisation techniques that have been utilised in Africans, to understand NS, include targeted approaches such as Sanger sequencing (number of studies, n = 14). While these studies did not satisfy our inclusion criteria, it is worth mentioning that these techniques have uncovered variants in genes important in NS such as *NPHS1*, *NPHS2*, *WT1*, and *APOL1*, leading us to hypothesise that there could be more variants (possibly undiscovered) that could be underlying NS in African populations. Hence, non-targeted approaches such as NGS techniques could offer more insights. Using NGS techniques, although currently limited to a few studies (n = 5) which have limited samples sizes, our review shows that there are other genes and/or variants that are implicated in NS and its different phenotypes (Table 3, Supplementary Tables S1 and S2). For example, *NPHS2* seems to be the mostly mutated gene in NS, with more variants even linked to other NS phenotypes (SRNS and FSGS) among Egyptian and Tunisian populations. These studies have only scratched the surface, and more remains to be performed in other African populations [13,27,31,39].

To determine whether this scarcity of genetic studies investigating the genetic spectrum of NS was unique to Africa or reflected a broader global trend, we conducted a secondary search excluding ‘Africa’ for comparison. The initial search, which included ‘Africa’ and related terms, were included in our search; we identified only 12 studies, with just three directly relevant to the application of WES in Africa and the molecular diagnosis and management of NS (Figure 2). The exclusion of ‘Africa’ from the search yielded 548 citations, 270 of which met the initial eligibility criteria before screening. While further exclusions would have been expected during screening, the number of relevant studies would still be considerably higher than in our Africa-specific search. Despite the limited number of studies retrieved and included in this review, with only three using WES to identify pathogenic variants, the power of WES in uncovering population-specific NS genetic variants has been reported. This is supported by the higher diagnostic yield in the Egyptian study that utilised WES (65.2%, n = 56) compared to the one that utilised WGS (57.4%, n = 47), although this difference might not be statistically significant as the sample sizes differ. Of the studies included in this review, three identified novel variants specific to African populations and are mostly predicted to be pathogenic (Table 3) [17,22,24]. Although they associated these variants with varying clinical outcomes, including treatment responses, further validation through functional assays to confirm causality is essential [17,22,24].

Bierzynska et al. [18] reported on the greatest number of likely pathogenic or pathogenic variants and that could be because that study had a larger sample size, included more genes in the panel, and had mixed population cohort than the other two studies [17,22]. They did not, however, report on the ethnicity of the participants the pathogenic variants were identified in; however, they do report on the observations from their Black participants (which they define as African or Afro-Caribbean) [18]. Similarly, the study conducted in France, although it had a bigger sample size and potentially identified more likely pathogenic or pathogenic variants, did not highlight which of the variants were novel or which were specific to particular population groups [23]. The WES results of three out of 10 participants were negative for variants in the ‘NS-related genes’ [18]. Notably, the participants in the study harboured the *APOL1 G1* and *G2* high-risk variants in either a homozygous or compound heterozygous state, and these participants presented with FSGS within the first 10 years of life [18]. This is consistent with the results obtained in a South African study by [39], which observed that about 70% of their steroid-sensitive FSGS participants carried the *APOL1 G1* high-risk variant, while *NPHS2* variants were absent. In this study, they identified that the presence of either or both *APOL1* risk alleles was associated with FSGS, although the frequencies of the alleles were lower than those observed in African Americans and in West Africa [8,40]. Gribouval et al. [23] identified an *APOL1* high-risk genotype (defined as the combination of *G1* and *G2* alleles) in 27.3% of their FSGS study participants (n = 77) originating from Africa, which was lower than the 60% identified in the participants originating from the French West Indies [23]. This study also investigated duplication at the *APOL1* locus, which includes *APOL1*, *APOL2*, and a part of *MYH9* and has been associated with increased susceptibility to CKD [41]. Although this duplication was identified in three families, none had severe phenotypes compared to the other participants [23].

It is, however, noteworthy that these *APOL1* risk variants are one of the most studied variants in Africa and have been strongly associated with an increased prevalence of FSGS in African Americans [42,43] and an increased risk of CKD, with frequencies particularly higher in West Africa [8,40] compared to Southern Africa. More evidence of the differences in the frequencies of *APOL1* risk variants across the African continent is highlighted in a study by Asharam et al. [13], where there was no statistically significant association of the high-risk alleles with NS, though this observation was likely due to their small sample size. Despite these disparities which can be attributable to geographical differences and the different trypanosome selection pressures related to the prevalence of *Trypanosoma brucei rhodesiense* and *Trypanosoma brucei gambiense* in Africa [42], screening for these *APOL1* in NS/FSGS diagnosis can lead to improved disease management and treatment.

Furthermore, the *NPHS2 V260E* variant, first identified in a study including North Africans and Europeans [44], was later identified in South African children of African descent (not in European or Indian South Africans) and was further confirmed to predict SRNS in South African children of African descent, especially those presenting with FSGS as a histopathological feature [13,17,39]. Due to the higher prevalence of SRNS in Africa and its nature of having non-favourable treatment outcomes, studies in Africa have focused on understanding the genetics of SRNS. Thus, to try and shed light on the genetic landscape of NS in SA, we performed WES in 56 SRNS patients and 29 controls (88% of which were Black). The WES results revealed that 17% of the black SRNS cases carried heterozygous putative mutations in the *INF2*, *CD2AP*, and *TRPC6* genes known to have autosomal dominant inheritance patterns (Table 2 and Table 3) [17]. Furthermore, a novel mutation predicted to be possibly damaging by PolyPhen (Supplementary Table S2) was identified in one SRNS case of Indian descent. It is noteworthy that in this study [17] the SSNS cases had no mutations in any of the investigated genes. However, analysing the additional genes associated with SRNS could have potentially revealed mutations in other genes or regions of the exomes, potentially increasing the diagnostic rate from the 39%. The limitation of the studies [13,39] that did not employ WES in their methods is that they could only confirm known variants associated with NS and could only identify novel variants within the gene region being studied, thereby limiting their identification of more population specific mutations.

Although mutations were not identified in all genes, the inclusion of the 33 SRNS genes in their WES gene panel yielded a diagnostic rate of 64.4% in Egyptian children [22]. Their WES data revealed a high association between CNS and *NPHS1* variants, similar to the Finnish population [45], while *NPHS2* variants were mainly found in non-congenital cases [22]. Similarly, we identified Egyptian founder mutations *NPHS2 M1L* and N199Kfs*14 previously identified in the study conducted on Egyptian children [34]. Though none of their early onset patients had the N199Kfs*14 [22], a different Egyptian study, which also reported the variant as having a founder effect, showed that patients with this variant presented with proteinuria even after two years of age, consistent with previous reports [38]. Furthermore, the *NPHS2 M1L* founder variant was identified in 57% of their study participants, with two of the cases presenting with CNS. This emphasises the distribution of NS, which is region-, population-, and ethnicity-specific, thus highlighting the need for genetic testing when diagnosing the different forms of NS to improve management, differing from previously reported studies conducted outside of the country, as highlighted in their paper [22]. The importance of genetic testing and WES molecular diagnosis is evident in this study because, despite majority of their participants exhibiting initial response to prescribed immunosuppressants, they later developed multi-drug resistance with their biopsies revealing a progression to glomerulosclerosis [22]. A change from MCD to FSGS has been previously reported and has been strongly associated with SRNS/FSGS-related variants; thus, cases can be screened to predict disease outcomes, as SRNS/FSGS has been reported to have the worst outcomes, with patients of reaching ESKD within the first 10 years of life [4].

## 5. Limitations

The studies included in this review are few (n = 3), and their sample sizes were limited. With the study conducted in the UK having a sample size of 187 participants [18], the South African study with 56 participants [17], and the Egyptian study [22] with 58 participants [22], this challenges the advocation for NGS utility in Africa for the diagnosis and management of NS in the continent. It is also important to note that, as much as the preliminary evidence suggests, WES may contribute to a better understanding of the genetic landscape of NS, the lack of representativeness of African populations in research, as highlighted in this review, calls for further research to be conducted. As mentioned, the UK and France studies [18,23] do not state which participants the mutations were identified in; therefore, the results reported in these studies might not exactly contribute to the ‘Africa’-specific variants but do highlight the population differences, as some of the variants in the known genes they reported have not been observed in continental African studies.

## 6. Presenting a Strong Call for Utilisation of WES and Concluding Remarks

Although WES has proven to be effective in identifying population specific pathogenic variants, Africa is still lagging in exploiting these benefits. This difference is reflected in the higher number of NS-related pathogenic variants reported in Western countries compared to those found in Africa (Figure 1). Along with the small number of studies identified in our review and the findings from [20], this points to a limited understanding of the genetic basis of NS across the African continent. The rapid discovery of monogenic causes and genetic risk factors for SRNS has greatly influenced its diagnosis and treatment in both the African and non-African populations. This progress is evident in the use of genetic information to make accurate, mechanism-based diagnoses and to develop new diagnostic tools [46,47,48]. Furthermore, in Western countries specifically, the use of WES in NS has led to significant improvements in patient outcomes [49]. Precision diagnostics enabled by WES allow for more accurate identification of monogenic causes of SRNS. This has had a direct impact on treatment strategies by helping clinicians avoid ineffective therapies, such as prolonged use of immunosuppressive drugs, which are often not effective for genetically caused NS [49]. Additionally, the ability to accurately diagnose the genetic causes of NS offers important prognostic information, enabling clinicians to tailor treatment plans to the specific needs of each patient, improving long-term outcomes. Understanding the genetic underpinnings of a patient’s condition allows healthcare providers to inform families about potential risks for siblings or future children, thus facilitating early intervention and surveillance. Moreover, WES has contributed to the discovery of new therapeutic targets, further enhancing the development of personalised treatments for NS. These advancements have also led to more cost-effective care (in the long term, despite the high initial costs of sequencing). By reducing misdiagnoses and unnecessary treatments, WES ultimately helps lower overall healthcare expenses [22,50,51].

The implementation of WES for NS in Africa seems to face significant challenges rooted in infrastructure, cost, and capacity. Many African countries lack the necessary laboratory facilities and sequencing equipment, which are essential for conducting WES [20]. Additionally, the costs of acquiring and maintaining such technology are prohibitively high, particularly when healthcare budgets are stretched to prioritise more immediate, life-saving interventions. The scarcity of trained bioinformaticians and genomics professionals further complicates the analysis and interpretation of WES data (even more WGS), as specialised skills are required to process and understand complex genetic information. As a result, most healthcare systems gravitate towards less costly diagnostic tests for NS (i.e., biochemical tests and kidney biopsies) averaging between USD 4–USD 60 per test [52,53] compared to genomic tests (i.e., WES) costing as high as USD 1000 per test (2020 costs) [54].

Another barrier is the underrepresentation of African populations in global genomic databases, which leads to challenges in variant interpretation (most relevant variants can be lost in in silico prediction) [17], because genetic variants common in African populations may not be well-documented or understood, hindering accurate diagnosis [17] as well as limiting the effects of WES. Evidently this was the case when WES was negative for four cases with positive family histories of NS [22]. Additionally, the lack of genomic literacy among healthcare professionals heightens the difficulty in utilising genomic data for clinical decision-making, limiting the integration of WES into routine clinical care. Within the African setting, there are no multidisciplinary collaborations between clinicians and scientists, as there is no connection between diagnosis and research, which in turn hinders the implementation of research findings within the clinical setting. The lack of genetic counsellors and the limited availability of genetic counselling training in Africa (currently offered by two South African universities and one Ghanian one) also create a significant barrier to the integration of genomic medicine into healthcare systems across Africa. Another important aspect that may hinder the implementation of WES is that there are currently limited African regulations or guidelines and standard operating procedures around genomic testing, and countries are cautious about approving genomics and biobanking studies [55]. Particularly in South Africa, there are no clear guidelines on research implementation into the clinical diagnostic setting. The African Society of Human Genetics together with H3Africa, is lobbying towards equity in genomics.

As NGS technology continues to evolve, there is a need for more tailored strategies to incorporate these advancements into healthcare systems, especially in resource-limited settings [50,51]. However, significant efforts are also needed to address the barriers to WES implementation [50,51]. Investments in infrastructure, training or mentorship, and research inclusion are critical to improving access to genomic medicine and ensuring that its advantages can be extended to African populations. It is important that policy makers also start to play an active role including mobilisation of funds to researchers working on such high priority areas [56] as well as foster collaborations with multiple stakeholders. Currently, there are initiatives such as the KidneyGen Africa and H3Africa that have been launched and work towards leveraging the lack of representation of Africa and its people in genomic research, but more can still be performed, particularly in terms of access to funding. This will lead to increased representation of African populations in genomic research and developing strategies that are tailored to the unique challenges of the continent, such as streamlining diagnostic processes and reducing the dependency on expensive bioinformatics tools. By addressing these challenges, WES could potentially transform the diagnosis and management of NS in Africa, offering a more precise, personalised approach to patient care.

## Figures and Tables

**Table 1:** Description of NS phenotypes.

NS Type	Presentation of NS Type
	Response to Treatment:
SSNS	Respond well to conventional corticosteroid therapy with a favourable long-term prognosis
SRNS	Experience partial, late, or complete resistance to corticosteroid treatment, thus experiencing frequent relapses and require alternative immunosuppressants.
	Age of Disease Manifestation:
CNS	From birth to 3 months
INS	From 3 months to 12 months
Childhood NS	From 12 months and beyond
	Histopathological Feature:
FSGS	Identified by podocyte depletion; a reduction of 20–40% in podocyte count results in segmental scarring of the glomeruli, leading to enlargement of the glomerulus and subsequent additional podocyte loss
MCD/MCNS	Distinguished by reversible alterations in podocyte architecture without significant podocyte loss
GN	Inflammation of the glomeruli
MN	Characterised as an autoimmune disorder that facilitates the accumulation of immune proteins within the glomerular basement membrane of the kidneys.
MDS	Characterised by sclerosis of the mesangial matrix resulting in minimal or no cell proliferation.

SSNS—Steroid-Sensitive Nephrotic Syndrome, SRNS—Steroid-Resistant Nephrotic Syndrome, CNS—Congenital Nephrotic Syndrome, INS—Infantile Nephrotic Syndrome, FSGS—Focal Segmental Glomerulosclerosis, MCD—Minimal Change Disease/MCNS—Minimal Change Nephrotic Syndrome, Glomerulonephritis, MN—Membranous nephropathy, MDS—Diffuse Mesangial Sclerosis. MCD, FSGS, and MN are the most common.

**Table 2:** Summarising the characteristics of the retrieved studies.

Population or Country	Sample Size	Age Range	NS Phenotypes	NGS Platform Used	Coverage Metrics	Genes with High Frequency of Mutations/Commonly Mutated Genes in NS Reported	Pathogenicity Tools Used	Diagnostic Yield (%)	References
South African	56	60–100 months	SRNS-FSGS	WES LC Sciences (Houston, TX, USA): Illumina HiSeq4000	Paired-end sequencing: average sequencing depth of >80×	*NPHS2*, *INF2*, *CD2AP* and *TRPC6*	PolyPhen-2 and SIFT	17	[17]
Egyptian	58	<2 years	NS, asymptomatic proteinuria	WES N/A	N/A	*NPHS1*, *NPHS2* and *PLCE1*	Mutation Taster, SIFT and PolyPhen	64.4	[22]
United Kingdom of Great Britain and Northern Ireland	187	<19 years	INS, primary or secondary SRNS	WES Illumina HiSeq	100 paired-end sequencing: average sequencing depth ~100×	*NPHS1*, *WT1* and *NPHS2*	MutPred, SIFT, Mutation Taster and Alamut splicing predictions.	26.2	[18]
France	152	0–64 years	SRNS and/or FSGS	NGS gene panel Multiplicom	N/A	*APOL1*, *NPHS1* and *NPHS2*	Mutation Taster, SIFT and PolyPhen2	11.2	[23]
Egyptian	47	7 months–22 years	SRNS	WGS IlluminaNovaSeq 6000	30×	*NPHS2*, *NPHS2* and *WT1*	Revel, CADD, SpliceAI	57.4	[24]

SRNS—Steroid resistant nephrotic syndrome; FSGS—Focal segmental glomerulosclerosis; NS—Nephrotic syndrome; INS—Infantile nephrotic syndrome; WES—Whole exome sequencing; N/A—not applicable as not provided in the study; NGS—Next-generation sequencing; WGS—Whole genome sequencing; SIFT—Sort intolerant from tolerant; CADD—Combined annotation dependent depletion.

**Table 3:** Novel variants identified in studies conducted on African populations with their predicted effects.

Gene	Variant	Predicted Effects	Genome Assembly	Type of NS	References
*TRPC6*	NM_004621.6:c.485G>T p.(Gly162Val)	Pathogenic Moderate	Hg19	SR-FSGS	[17] *
*INF2*	ENST00000252527.8:c.1226A>C p.(Lys409Thr)				
*PLCE1*	NM_016341.4:c.3194C>A p.(Ala1065Glu)	Benign Supporting			
*ACTN4*	ENST00000390009.3:c.174C>T p.(Ala58=)	Benign Strong			
*PLCE1*	NM_001288989.2:c.689_690del p.(Tyr230CysfsTer6)	Pathogenic	-	NS	[22] **
	NM_016341.4:c.5364C>G p.(Tyr1788Ter)	Likely Pathogenic		SRNS, Axenfeld-Rieger syndrome	
*COL4A3*	NM_000091.5:c.820_821delGGinsC p.(Gly274HisfsTer49)	Likely Pathogenic		Isolated SRNS	
*OSGEP*	NM_017807.4:c.775A>T p.(Ile259Phe)	Likely Pathogenic		Pierson and Galloway Mowart syndromes and adrenalin insufficiency	
*LAMB2*	NM_002292.4:c.5368C>T p.(Gln1790Ter)	Likely Pathogenic			
*SGPL1*	NM_003901.4:c.1013A>G p.(Asp338Gly)	Likely Pathogenic			
*PAX2*	NM_003989.5:c.869del p.(Pro290LeufsTer16)	Pathogenic		Multicystic dysplastic kidney	
*NPHS1*	Deletion of exon 8	-	Hg19	N/A	[18] ***
	NM_004646.4:c.2387G>A p.(Gly796Glu)	Likely Pathogenic		Diffuse mesangial sclerosis (DMS)	
	NM_004646.4:c.136G>T p.(Gly46Trp)	Uncertain Significance		N/A	
	NM_004646.4:c.925G>A p.(Glu309Lys)	Uncertain Significance			
	NM_004646.4:c.1584C>T p.(Cys528=)	Benign Moderate		CNS	
	NM_004646.4:c.1910_1912del p.(Phe637del)	-		N/A	
*NPHS2*	NM_014625.4:c.156del p.(Thr53ProfsTer46)	Pathogenic/Likely Pathogenic		N/A	
	NM_014625.4:c.378+2_378+3del p.?	Likely Pathogenic		SRNS	
*ACTN4*	NM_001322033.2:c.779_787del p.(Tyr260_Ser262del)	-		FSGS	
*TRPC6*	NM_004621.6:c.523C>T p.(Arg175Trp)	Pathogenic/Likely Pathogenic		FSGS	
*MYO1E*	NM_004998.4:c.2094T>A p.(Tyr698Ter)	-		N/A	
*DGKE*	ENST00000284061.3:c.1303C>T p.(Arg435Ter)	-		N/A	
*LMX1B*	NM_002316.4:c.676C>T p.(Leu226Phe)	Pathogenic Strong/Uncertain Significance		N/A	
*COL4A5*	NM_033380.3:c.3097G>C p.(Gly1033Arg)	Pathogenic Moderate		N/A	
*ADCK4/ COQ8B*	NM_024876.4:c.101G>A p.(Trp34Ter)	Benign Moderate		SRNS	
	NM_024876.4:c.954_956dup p.(Thr319dup)	-			
*CRB2*	NM_173689.7:c.3089_3104dup p.(Gly1036AlafsTer43)	Pathogenic/Likely Pathogenic		SRNS	
*PODXL*	NM_005397.4:c.1427A>T p.(His476Leu)	Pathogenic Moderate		N/A	
*OCRL*	NM_001587.4:c.1467-2A>G p.?	Pathogenic Strong		N/A	
*COL4A3*	NM_000091.5:c.2126-1G>A	Likely Pathogenic	Hg38	SRNS	[24] **
*MYO1E*	NM_004998.4:c.1616+1G>C	Likely Pathogenic			
*NPHS1*	NM_004646.4: c.2758T>C p.(Cys920Arg)	Likely Pathogenic			
*NPHS2*	NM_014625.4:c.596dup p.(Asn199LysfsTer14)	Likely Pathogenic			
*NUP93*	NM_014669.5:c.554 A>G p.(Tyr185Cys)	Variant of Uncertain Significance			
*PLCE1*	NM_016341.4:c.2779G>T p(.Gly927Ter)	Likely Pathogenic			
*PODXL*	NM_001018111.3:c.1101+2T>C	Likely Pathogenic			
*SMARCAL1*	NM_014140.4:c.1096+4A>G	Variant of Uncertain Significance			
*WT1*	NM_024426.6:c.700G>C p.(Gly234 Arg)	Variant of Uncertain Significance			

* In silico prediction on VarSome [25], ** ACMG classification reported in paper [22], *** ClinVar clinical significance [26].

## Data Availability

No new data were created or analyzed in this study. Data sharing is not applicable to this article.

## Supplementary Materials

The following supporting information can be downloaded at: https://www.mdpi.com/article/10.3390/genes16111295/s1, Table S1: Papers retrieved during literature search and reasons for exclusion/inclusion in the literature review [57,58,59,60,61,62,63,64,65,66]. Table S2: Gene-disease association evidence levels according to ClinGen’s Glomerulopathy Gene Curation Expert Panel. Table S3: Variants identified across the three studies with complete HGVS nomenclature, population frequencies from gnomAD database where available, and functional predictions from multiple in silico tools.
