# Supplementary material for: Whole-Exome Sequencing for Molecular Diagnosis of Paediatric Nephrotic Syndrome in Africa: A Call for Implementation
Source: Genes (Basel). 2025 Oct 31;16(11):1295. doi: 10.3390/genes16111295 (PMC12652124; doi:10.3390/genes16111295)
Supplement: Supplementary file 1 [file genes-16-01295-s001.zip › genes-3843318-supplementary.pdf]

Supplementary Table S1: Papers retrieved during literature search and reasons for exclusion/inclusion in the literature review

Title	Exclusion/Inclusion	Database	Reference
Clinical and genetic heterogeneity in familial steroid-sensitive nephrotic syndrome	Excluded (non-African participants)	PubMed	[57]
Causal and putative pathogenic mutations identified in 39% of children with primary steroid-resistant nephrotic syndrome in South Africa	Included		[17]
Genomic and clinical profiling of a national nephrotic syndrome cohort advocates a precision medicine approach to disease management	Included		[18]
Recessive <i>NOS1AP</i> variants impair actin remodelling and cause glomerulopathy in humans and mice	Excluded (non-African participants)		[58]
A single-gene cause in 29.5% of cases of steroid-resistant nephrotic syndrome	Excluded (non- African participants)		[34]
Rapid detection of monogenic causes of childhood-onset steroid-resistant nephrotic syndrome.	Excluded (Outside the year range)		[59]
Genomic and clinical profiling of a national nephrotic syndrome cohort advocates a precision medicine approach to disease management	Excluded (Duplicate)		[18]
Causal and putative pathogenic mutations identified in 39% of children with primary steroid-resistant nephrotic syndrome in South Africa	Excluded (Duplicate)	Medline	[17]

Genetic stratification reveals *COL4A* variants and spontaneous remission in Egyptian children with proteinuria in the first 2years of life	Included		[22]
Genomic and clinical profiling of a national nephrotic syndrome cohort advocates a precision medicine approach to disease management	Excluded (Duplicate)		[18]
NPHS2 V260E Is a Frequent Cause of Steroid-Resistant Nephrotic Syndrome in Black South African Children	Excluded (Not NGS)		[13]
APOL1 risk genotype in European steroid-resistant nephrotic syndrome and/or focal segmental glomerulosclerosis patients of different African ancestries.	Included		[23]
Prospects of genetic testing for steroid-resistant nephrotic syndrome in Nigerian children: a narrative review of challenges and opportunities	Excluded (Literature review)		[60]
Whole genome sequencing identifies monogenic disease in 56.1% of families with early-onset steroid-resistant nephrotic syndrome.	Included		[24]
Detailed Pathophysiology of Minimal Change Disease: Insights into Podocyte Dysfunction, Immune Dysregulation, and Genetic Susceptibility	Excluded (Literature Review)	Scopus	[61]
Fertility Preservation and Restoration for Patients with Complex Medical Conditions	Excluded (Book/Not NS or NGS)		[62]

Diagnostic Pathology: Kidney Diseases: A volume in Diagnostic Pathology	Excluded (Book/ Not NGS)	[63]
Effects of a novel ANLN E841K mutation associated with SRNS on podocytes and its mechanism	Excluded (non-African population)	[64]
267 Spanish Exomes Reveal Population-Specific Differences in Disease-Related Genetic Variation	Excluded (non-Africa population)	[65]
Changing epidemiology of nephrotic syndrome in Nigerian children: A cross-sectional study	Excluded (Not NGS, Clinical and Epidemiological)	[66]
NPHS2 V260E Is a Frequent Cause of Steroid-Resistant Nephrotic Syndrome in Black South African Children	Excluded (Duplicate, not NGS)	[13]

Supplementary Table S2: Gene-disease association evidence levels according to ClinGen's Glomerulopathy Gene Curation Expert Panel

Gene	No. of studies identified in	Gene-Disease Validity Classification	GennCC Classification$^{\alpha}$
TRPC6	3	Under review	Strong, Supportive
INF2	2	Definitive	-
PLCE1	4	Definitive	-
ACTN4	2	Pre-curation	Moderate, Supportive, Strong
COL4A3	3	Definitive	-
OSGEP	1	In scope	Strong, Supportive
LAMB2	4	Pre-curation	Definitive, Strong, Supportive
SGPL1	1	Definitive	-
PAX2	1	Definitive	-
NPHS1	4	Definitive	-
NPHS2	5	Pre-curation	Definitive, Strong, Supportive, Limited
MYO1E	2	Under review	Strong, Supportive
DGKE	1	Definitive	-
LMX1B	3	Definitive	-
COL4A5	2	Definitive	-
ADCK4/ COQ8B	1	Definitive	-
CRB2	1	Definitive	-

PODXL	2	In scope	-
OCRL	1	Definitive	-
CD2AP	2	Definitive	-
SMARCAL1	1	In scope	Definitive, Strong, Supportive
WT1	4	Pre-curation	Definitive, Strong, Supportive
CUBN	1	In scope	Definitive, Supportive, Strong
PDSS2	1	In scope	Definitive, Supportive, Strong, Moderate
SYNPO2	1	Pre-curation	-
NUP93	2	Definitive	-

α The Gene Curation Coalition

Supplementary Table S3: Variants identified across the three studies with complete HGVS nomenclature, population frequencies from gnomAD database where available, and functional predictions from multiple in silico tools.

**Gene**	**Variant**[α]	**Insilico Predicted Effects**				**gnomAD Frequency**
		**PolyPhen-2**	**SIFT**	**MT**	**MutPred**	
ACTN4	ENST00000390009.3:c.174C>T p.(Ala58=)	N/A	N/A	-	-	0.0000239
	NM_001322033.2:c.779_787del p.(Tyr260_Ser262del)	-	N/A	N/A	N/A	N/A
ADCK4/ COQ8B	NM_024876.4:c.101G>A p.(Trp34Ter)	-	N/A	N/A	N/A	N/A
	NM_024876.4:c.954_956dup p.(Thr319dup)		N/A	N/A	N/A	N/A
COL4A3	NM_000091.5:c.820_821delGGinsC p.(Gly274HisfsTer49)	N/A	N/A	N/A	-	N/A
COL4A5	NM_033380.3:c.3097G>C p.(Gly1033Arg)	-	High Risk	Tolerated	Deleterious	N/A
CRB2	NM_173689.7:c.3089_3104dup p.(Gly1036AlafsTer43)	-	N/A	N/A	N/A	0.000916
DGKE	ENST00000284061.3:c.1303C>T p.(Arg435Ter)	-	N/A	N/A	N/A	N/A
INF2	ENST00000252527.8:c.1226A>C p.(Lys409Thr)	Benign	Tolerated	-	-	N/A
LAMB2	NM_002292.4:c.5368C>T p.(Gln1790Ter)	N/A	N/A	N/A	-	N/A
LMX1B	NM_002316.4:c.676C>T p.(Leu226Phe)	-	Low Risk	Deleterious	Deleterious	N/A
MYO1E	NM_004998.4:c.2094T>A p.(Tyr698Ter)	-	N/A	N/A	N/A	N/A
NPHS1	Deletion of exon 8	-	N/A	N/A	N/A	N/A
	NM_004646.4:c.2387G>A p.(Gly796Glu)		Low Risk	Deleterious	Deleterious	N/A
	NM_004646.4:c.136G>T p.(Gly46Trp)	-	High Risk	Deleterious	Deleterious	N/A
	NM_004646.4:c.925G>A p.(Glu309Lys)		Medium Risk	Tolerated	Deleterious	0.00000405

	NM_004646.4:c.1584C>T p.(Cys528=)	-	N/A	N/A	N/A	N/A
	NM_004646.4:c.1910_1912del p.(Phe637del)	-	N/A	N/A	N/A	N/A
NPHS2	NM_014625.4:c.156del p.(Thr53ProfsTer46)	-	N/A	N/A	N/A	N/A
	NM_014625.4:c.378+2_378+3del p.?	-	N/A	N/A	N/A	N/A
OCRL	NM_001587.4:c.1467-2A>G p.?	-	Likely*	N/A	N/A	N/A
OSGEP	NM_017807.4:c.775A>T p.(Ile259Phe)	Probably Damaging	Deleterious	Disease Causing	-	N/A
PAX2	NM_003989.5:c.869del p.(Pro290LeufsTer16)	N/A	N/A	N/A	-	N/A
PLCE1	NM_016341.4:c.3194C>A p.(Ala1065Glu)	Benign	Tolerated	-	-	N/A
	NM_001288989.2:c.689_690del p.(Tyr230CysfsTer6)	N/A	N/A	NA	-	N/A
	NM_016341.4:c.5364C>G p.(Tyr1788Ter)	N/A	N/A	N/A	-	
PODXL	NM_005397.4:c.1427A>T p.(His476Leu)	-	Medium Risk	Deleterious	Deleterious	N/A
SGPL1	NM_003901.4:c.1013A>G p.(Asp338Gly)	Probably Damaging	Deleterious	Disease Causing	-	N/A
TRPC6	NM_004621.6:c.485G>T p.(Gly162Val)	Probably damaging	Damaging	-	-	N/A
	NM_004621.6:c.523C>T p.(Arg175Trp)	-	Deleterious	Deleterious	Medium Risk	N/A

-:Studies did not use the prediction tool, N/A: prediction tool used but yielded no results/ data not available, *:reported in study without classification. $^{\alpha}$ **other variants reported which were not novel are found in** [17,18,22–24].
